# Sociodemographic and health-related predictors of self-reported mammogram, faecal occult blood test and prostate specific antigen test use in a large Australian study

**DOI:** 10.1186/1471-2458-13-429

**Published:** 2013-05-03

**Authors:** Marianne F Weber, Michelle Cunich, David P Smith, Glenn Salkeld, Freddy Sitas, Dianne O’Connell

**Affiliations:** 1 Cancer Research Division, Cancer Council NSW, PO Box 572, Kings Cross, Sydney, NSW 1340, Australia; 2 School of Public Health, The University of Sydney, Sydney, NSW 2006, Australia; 3 School of Medicine and Public Health, The University of Newcastle, Newcastle, NSW 2308, Australia; 4 School of Public Health and Community Medicine, The University of New South Wales, Kensington, NSW 2052, Australia

**Keywords:** Cancer screening, Mammography, Faecal occult blood test, Prostate specific antigen test, Sociodemographic characteristics, Socioeconomic status

## Abstract

**Background:**

While several studies have examined factors that influence the use of breast screening mammography, faecal occult blood tests (FOBT) for bowel cancer screening and prostate specific antigen (PSA) tests for prostate disease in Australia, research directly comparing the use of these tests is sparse. We examined sociodemographic and health-related factors associated with the use of these tests in the previous two years either alone or in combination.

**Methods:**

Cross-sectional analysis of self-reported questionnaire data from 96,711 women and 82,648 men aged 50 or over in The 45 and Up Study in NSW (2006–2010).

**Results:**

5.9% of men had a FOBT alone, 44.9% had a PSA test alone, 18.7% had both tests, and 30.6% had neither test. 3.2% of women had a FOBT alone, 56.0% had a mammogram alone, 16.2% had both and 24.7% had neither test. Among men, age and socioeconomic factors were largely associated with having both FOBT and PSA tests. PSA testing alone was largely associated with age, family history of prostate cancer, health insurance status and visiting a doctor. Among women, age, use of hormone replacement therapy (HRT), health insurance status, family history of breast cancer, being retired and not having a disability were associated with both FOBT and mammograms. Mammography use alone was largely associated with age, use of HRT and family history of breast cancer. FOBT use alone among men was associated with high income, living in regional areas and being fully-retired and among women, being fully-retired or sick/disabled.

**Conclusions:**

These results add to the literature on sociodemographic discrepancies related to cancer screening uptake and highlight the fact that many people are being screened for one cancer when they could be screened for two.

## Background

Prostate, breast and bowel cancer are the most commonly diagnosed cancers in Australia and apart from lung cancer, also account for the highest number of cancer-related deaths [1]. There is clear evidence that early detection of breast and bowel cancer via screening reduces the mortality associated with these diseases [2-4] and Australia has national, government-funded screening programs for both these cancers (breast since 1992 and bowel since 2006). In contrast, although a mortality benefit has been found for the early detection of prostate cancer using prostate specific antigen (PSA) testing [5], this form of screening is not recommended by the Australian Government because the harms outweigh the benefits [5,6]. However, PSA tests have been subsidised by the Australian Government since 1989 and PSA testing for the early detection of prostate cancer has received widespread media attention in recent years [7]. Thus, PSA testing is effectively used as a *de facto,* albeit disorganised, prostate cancer screening program [8].

Among Australian women, participation in the breast cancer screening program is around 55% (in the target age group of 50–69 years) [9]. However, screening mammograms are also commonly accessed through the private sector on a user-pays basis and so the participation rate is more likely to be around 75% [10,11]. In the bowel cancer screening program the participation rate among those invited is around 38.4% (currently only people turning 50, 55 and 65 are invited via a mailed, faecal occult blood test; FOBT) [12]. FOBTs are also available outside the program, but are not widely used and so the overall participation rate in the target age group remains similar to the program participation rate [10]. The prevalence of PSA testing for prostate cancer screening purposes in Australia is unknown but estimated to be quite high. A nationally representative study ten years ago found that 63% of men aged >50 years had ever had a PSA test [13] and this proportion is likely to have risen significantly since then [8,14].

While there have been a few recent studies examining factors that influence mammography, FOBT and PSA test use in Australia [11,14-19], research comparing the use of these different cancer screening tests is sparse. Several studies in the USA have directly compared factors associated with being screened with more than one of these test types [20-25] but only one study (limited to men) has been reported from Australia [26]. These studies have all found that many people are screened for breast and prostate cancer but not for bowel cancer. Although the test types are very different in nature (FOBT is self-administered, mammography is an imaging technique done in a specialist clinic and PSA tests are blood tests ordered by a general practitioner), screening for one cancer type may potentially be a “teachable moment” for screening for another cancer type [27-29].

The objective of our study was to examine factors related to the use of FOBT, mammography and PSA tests either alone or in combination. These factors included individual-level socio-economic indicators as well as demographic characteristics and health-related factors. We used self-reported, cross-sectional data from The 45 and Up Study in New South Wales (NSW; Australia’s most populous state) from participants aged 50 years and over, who reported that they had never had cancer. In a previous report, we found that 45 and Up Study participants were more than twice as likely to have a FOBT for bowel cancer if they had also had a mammogram or a PSA test [30]. By investigating the use of a range of cancer screening tests within the same population, commonalities and discrepancies in the determinants of cancer screening uptake can be more clearly identified.

## Methods

### Study sample

The 45 and Up Study is a population-based cohort study of people aged 45 and over in NSW [31]. The cohort was established with the aim of providing reliable evidence to inform health policy to support Australia’s healthy ageing population. Participants were randomly sampled from the Medicare Australia database, Australia’s universal health insurance system, which includes all citizens and permanent residents of Australia, some temporary residents and refugees. People aged 80 years and over and residents of regional areas were oversampled by a factor of two. Participants completed a mailed self-administered questionnaire and consent form. The participation rate was 18%, however The 45 and Up study sample has excellent heterogeneity and is reasonably representative of the NSW population; has a response rate comparable to similar studies internationally and in Australia; and is among the most representative large scale cohort studies in the world [32]. This paper uses the baseline cross-sectional data from 232,056 people aged 50 and over who completed the questionnaire between January 2006 and February 2010 (77% completed the questionnaire in 2008). We chose the lower age limit of 50 years because screening for bowel and breast cancer is not recommended for people younger than 50 if they are at normal risk, as is PSA testing for prostate cancer in some Australian guidelines (e.g. the Urological Society of Australia and New Zealand [33]).

Participants who reported ever having had cancer of any type (except non-melanoma skin cancer) were excluded from analysis because many of these individuals would have undergone more frequent surveillance than those without cancer, n = 39,897 (17%; 20,502 men and 19,395 women). An additional 7,003 men and 5,797 women were excluded because they failed to provide sufficient information on screening (6.6%). This left 82,648 men and 96,711 women for the main analyses (n = 179,359).

The 45 and Up Study was approved by the University of New South Wales Human Research Ethics Committee and the Cancer Council New South Wales Ethics Committee.

### Ascertainment of screening use

A self-reported history of bowel cancer screening was ascertained from the question, “Have you ever been screened for colorectal (bowel) cancer? If yes, please indicate which test(s) you had”. We restricted our results to FOBT use rather than colonoscopy or sigmoidoscopy because the National Bowel Cancer Screening Program uses FOBT tests, and colonoscopy/sigmoidoscopy can be used as a diagnostic test as well as a screening test. Breast cancer screening was ascertained from the question “Have you ever been for a breast screening mammogram?”. A history of PSA testing was ascertained from the question, “Have you ever had a blood test ordered by your doctor to check for prostate disease? (PSA test)”. We were not able to distinguish men who had a PSA test for prostate cancer screening from those who may have had a PSA test to investigate disease. However, approximately two thirds of the PSA tests administered Australia-wide in 2008 were for screening [34]. Additionally, we excluded men with a history of prostate cancer, which would have eliminated most men who had PSA tests to monitor disease and we compared the results from the full dataset with a sample of men that excluded those who reported ever having an enlarged prostate (16%). For all tests, respondents were asked to indicate how long ago (in years) they had used each test type and the analysis focussed on tests received in the previous two years.

### Ascertainment of socioeconomic, demographic and health characteristics

All factors were obtained from the self-administered questionnaire and are listed in Tables 1 and 2. These included age, education, annual household income from all sources (note, the mean annual Australian income in 2009–2010 for people aged 45 years or older was $50,490 [35]), married or living with a partner, language spoken at home, country of birth, need help with daily tasks due to illness or disability, psychological distress as measured by the Kessler 10 scale [36], and ever used hormone replacement therapy (HRT; women only). We also examined health insurance status, categorised as having 1) a health care concession card (subsidised care for low income earners), 2) a Department of Veterans’ Affairs card (subsidised care for current and past members of the Australian defence force and their spouses), 3) private health insurance without extras (covers ambulance and hospital services only), 4) private health insurance with extras (i.e. covers dental treatments and specialist services such as physiotherapy), or 5) no concession card or health Insurance. It should be noted, however, that all Australians have free universal access to hospital treatment in public hospitals and subsidised out-of-hospital medical treatment and medications. Those with concession cards receive larger subsidies and those with private health insurance are covered (to varying degrees, depending on the policy) for treatment in private hospitals.

**Table 1:** Characteristics of men in the 45 and Up study who reported ever having a faecal occult blood test (FOBT), prostate specific antigen (PSA) test, both tests, or neither test in the previous 2 years

	**n (%)**^**1**^	**% FOBT only**	**% PSA test only**	**% Both tests**	**% Neither test**
**Total n**	82,648 (100)	5.9	44.9	18.7	30.6
**Age**					
50-59	32102 (38.8)	7.1	40.3	16.7	35.9
60-69	27572 (33.4)	5.6	47.6	24.0	22.8
70+	22974 (27.8)	4.8	47.7	14.9	32.5
**Family history of cancer**					
Bowel	9449 (11.4)	6.4	46.3	17.6	29.7
Prostate	7173 (8.7)	4.5	50.4	23.0	22.1
Bowel and Prostate	1421 (1.7)	5.4	49.6	20.7	24.3
Other Cancer	15816 (19.1)	6.4	44.3	19.6	29.7
None	48789 (59.0)	5.9	43.8	17.8	32.4
Place of residence*					
Major City	38086 (46.1)	5.2	46.0	16.4	32.4
Inner Region	28464 (34.4)	6.3	44.5	20.5	28.7
Outer Region	14498 (17.5)	7.2	42.3	21.4	29.2
Remote or Very Remote	1535 (1.9)	4.7	46.5	14.9	33.9
**Highest qualification**					
No school certificate or other qualification	8876 (10.7)	4.7	45.7	13.8	35.8
School or intermediate certificate	12535 (15.2)	5.3	47.4	17.5	29.9
Higher school or leaving certificate	7932 (9.6)	5.6	44.3	16.6	33.6
Trade, apprenticeship, certificate or diploma	31105 (37.6)	5.9	45.7	19.4	29.0
University degree or higher	20849 (25.2)	7.1	42.0	21.4	29.5
**Employment status**					
Full time work	22345 (27.0)	6.7	42.8	17.5	33.0
Part time work	5873 (7.1)	6.5	44.3	20.0	29.3
Self employed	11526 (14.0)	5.9	45.1	18.1	30.8
Partially retired	3935 (4.8)	5.9	46.1	23.4	24.6
Fully retired	32971 (39.9)	5.4	46.5	20.2	27.9
Unemployed	1529 (1.9)	6.3	37.8	9.2	46.6
Unpaid work	646 (0.8)	6.2	45.2	15.2	33.4
Looking after home/family	394 (0.5)	5.8	41.9	11.7	40.6
Sick/disabled	2282 (2.8)	4.4	44.0	11.0	40.7
Other	674 (0.8)	3.9	45.6	15.7	34.9
**Income ($AUD)**^†^					
Less than 5,000	982 (1.2)	4.1	43.0	8.3	44.7
5,000-9,000	2953 (3.6)	5.2	43.8	9.2	41.8
10,000-19,000	11543 (14.0)	5.0	45.4	14.1	35.5
20,000-29,000	8545 (10.3)	5.8	46.2	18.4	29.5
30,000-39,000	7231 (8.8)	6.4	44.8	21.5	27.4
40,000-49,000	6745 (8.2)	6.0	44.2	21.7	28.2
50,000-69,000	9695 (11.7)	6.4	44.6	21.9	27.2
70,000 or more	22676 (27.4)	6.9	44.1	20.7	28.3
Prefer not to answer	10331 (12.5)	4.8	46.0	17.4	31.9
**Health insurance status**					
Health care concession card	14022 (17.0)	5.0	43.5	14.1	37.5
Department of Veterans’ Affairs card	1832 (2.2)	5.1	42.3	12.1	40.6
Private health insurance without extras	41033 (49.7)	6.2	46.6	22.0	25.3
Private health insurance with extras	12142 (14.7)	6.0	46.1	20.2	27.6
No concession card or health Insurance	12141 (14.7)	6.4	40.3	12.8	40.5
**Married or living with a partner**					
No	15150 (18.3)	5.7	40.9	13.4	40.0
Yes	66802 (80.8)	6.0	45.7	19.9	28.5
**Non-English language spoken at home**					
No	73979 (89.5)	6.1	44.8	19.7	29.3
Yes	8667 (10.5)	4.2	44.9	9.5	41.4
**Been treated by a doctor in the past month**^§^					
No	38102 (46.1)	6.5	41.5	18.5	33.5
Yes	35939 (43.5)	5.4	48.4	19.6	26.7
**Country of birth**					
Australia	59373 (71.8)	6.2	45.5	20.3	28.1
Other English speaking country	11214 (13.6)	6.4	41.2	17.8	34.6
Non-English speaking country	11222 (13.6)	4.3	45.4	11.0	39.2
**Need help with daily tasks due to illness or disability**					
No	76255 (92.3)	6.1	44.8	19.5	30.0
Yes	3761 (4.6)	4.3	43.8	11.9	40.0
**Psychological distress Level**					
Well	67398 (81.6)	6.1	44.7	19.7	29.5
Mild	3994 (4.8)	6.0	43.9	16.3	33.9
Moderate	1294 (1.6)	5.0	45.4	14.4	35.2
Severe	1243 (1.5)	5.2	41.8	12.6	40.5

^1^ Missing data for each covariate has been omitted for the sake of brevity.

* Using the mean Accessibility/Remoteness Index of Australia (ARIA+).

† Pre-tax annual household income from all sources.

§Treated for heart attack, other heart disease, high blood pressure, high blood cholesterol, blood clotting problems, asthma, osteoarthritis, thyroid problems, osteoporosis/low bone density, depression, or anxiety.

**Table 2:** Characteristics of women in the 45 and Up study who reported ever having a faecal occult blood test (FOBT), mammogram, both tests, or neither test in the previous 2 years

	**Total n (%)**^**1**^	**% FOBT only**	**% Mammogram only**	**% Both tests**	**% Neither test**
**Total n**	96711 (100)	3.2	56.0	16.2	24.7
**Age**					
50-59	42646 (44.1)	2.5	62.5	17.1	18.0
60-69	30538 (31.6)	2.0	65.4	21.7	11.0
70+	23527 (24.3)	5.9	32.0	7.3	54.7
**Family history of cancer**					
Bowel	11836 (12.2)	4.1	54.1	16.8	25.0
Breast	9937 (10.3)	2.3	62.5	18.1	17.1
Bowel and Breast	2077 (2.2)	3.0	58.7	17.4	20.9
Other Cancer	20273 (21.0)	2.9	57.1	17.2	22.8
None	52588 (54.4)	3.2	54.7	15.2	27.0
**Hormone replacement therapy use**					
Ever	40641 (42.0)	2.8	61.5	20.0	15.7
Never	53905 (55.7)	3.4	52.2	13.6	30.8
Place of residence*					
Major City	42402 (43.8)	2.9	55.8	14.3	27.0
Inner Region	34676 (35.9)	3.3	56.1	17.2	23.5
Outer Region	17697 (18.3)	3.3	55.9	18.8	22.0
Remote or Very Remote	1853 (1.9)	3.1	60.4	15.1	21.4
**Highest qualification**					
No school certificate or other qualification	12720 (13.2)	3.4	52.1	10.5	34.0
School or intermediate certificate	28347 (29.3)	2.9	57.0	15.7	24.5
Higher school or leaving certificate	9297 (9.6)	2.9	55.9	13.9	27.3
Trade, apprenticeship, certificate or diploma	24918 (25.8)	3.5	55.9	18.2	22.4
University degree or higher	19928 (20.6)	3.1	57.7	19.5	19.7
**Employment status**					
Full time work	16829 (17.4)	2.0	64.0	15.6	18.5
Part time work	16599 (17.2)	2.5	63.8	18.7	15.0
Self employed	6559 (6.8)	2.6	60.7	17.9	18.8
Partially retired	3030 (3.1)	2.9	59.3	22.1	15.8
Fully retired	39603 (41.0)	4.1	48.2	15.8	31.8
Unemployed	2088 (2.2)	3.0	54.6	9.4	33.1
Unpaid work	1952 (2.0)	2.9	59.8	17.0	20.3
Looking after home/family	5914 (6.1)	2.7	58.0	14.7	24.6
Sick/disabled	2004 (2.1)	3.8	54.9	9.2	32.0
Other	1245 (1.3)	2.8	56.0	12.4	28.8
**Income ($AUD)**^†^					
Less than 5,000	1878 (1.9)	3.0	52.6	10.8	33.7
5,000-9,000	4423 (4.6)	3.4	50.1	9.6	37.0
10,000-19,000	14983 (15.5)	4.3	48.1	12.1	35.6
20,000-29,000	9511 (9.8)	3.7	54.3	17.2	24.9
30,000-39,000	7593 (7.9)	3.3	58.6	18.5	19.7
40,000-49,000	6565 (6.8)	2.9	58.8	19.1	19.3
50,000-69,000	9214 (9.5)	2.7	60.4	19.3	17.6
70,000 or more	17348 (17.9)	2.3	62.5	19.6	15.7
Prefer not to answer	20589 (21.3)	3.1	57.1	16.0	23.8
**Health insurance status**					
Health care concession card	18482 (19.1)	3.7	49.0	11.2	36.0
Department of Veterans’ Affairs card	1095 (1.1)	4.8	32.8	6.6	55.9
Private health insurance without extras	47793 (49.4)	2.9	59.8	19.2	18.2
Private health insurance with extras	14187 (14.7)	3.2	56.2	18.0	22.6
No concession card or health Insurance	13428 (13.9)	3.1	54.8	12.1	30.1
**Married or living with a partner**					
No	28821 (29.8)	4.0	47.3	11.8	36.9
Yes	67599 (69.9)	2.8	59.7	18.0	19.5
**Non-english language spoken at home**					
No	88039 (91.0)	3.2	55.9	16.9	24.0
Yes	8671 (9.0)	2.5	56.7	8.6	32.2
**Country of birth**					
Australia	73198 (75.7)	3.2	56.2	17.2	23.4
Other English speaking country	11848 (12.3)	3.4	55.0	16.6	25.0
Non-English speaking country	10720 (11.1)	2.8	56.0	9.4	31.8
**Been treated by a doctor in the past month**§					
No	39055 (40.4)	2.7	58.6	17.0	21.8
Yes	49328 (51.0)	3.6	53.7	16.1	26.6
**Need help with daily tasks due to illness or disability**					
No	88048 (91.0)	3.1	57.3	16.9	22.8
Yes	5247 (5.4)	4.1	39.0	8.1	48.7
**Psychological distress Level**					
Well	74194 (76.7)	3.1	57.2	17.6	22.1
Mild	5366 (5.6)	3.2	57.9	14.1	24.8
Moderate	1706 (1.8)	2.9	53.8	13.0	30.3
Severe	1679 (1.7)	2.9	54.6	10.3	32.3

^1^ Missing data for each covariate has been omitted for the sake of brevity.

* Using the mean Accessibility/Remoteness Index of Australia (ARIA+).

† Pre-tax annual household income from all sources.

§Treated for heart attack, other heart disease, high blood pressure, high blood cholesterol, blood clotting problems, asthma, osteoarthritis, thyroid problems, osteoporosis/low bone density, depression, or anxiety.

The questionnaire also collected information about recent treatment for a number of medical conditions (heart attack, other heart disease, high blood pressure, high blood cholesterol, blood clotting problems, asthma, osteoarthritis, thyroid problems, osteoporosis/low bone density, depression and/or anxiety) and in the absence of a single question about visits to a doctor, these items were combined to get a partial measure of contact with the health system for reasons other than screening.

### Analyses

We examined the proportion of men who reported having a PSA test alone, a FOBT alone, neither test, or both tests within the past 2 years by socioeconomic, demographic and health characteristics. Similarly, we compared the proportion of women who reported having a mammogram alone, a FOBT alone, neither test or both tests within the last 2 years by socioeconomic, demographic and health characteristics.

Odds ratios and 95% confidence intervals (CI) corresponding to receiving each test type, both tests, or neither test for each socioeconomic, demographic and health factor were estimated using multinomial logistic regression with a generalised logit link function. Each model included all the factors listed in Table 1 for men and Table 2 for women. Missing values for each covariate were included in the models as a separate term to prevent loss of information (data not shown). We also analysed the data after excluding participants with missing values for any covariate.

## Results

### Descriptive statistics

Of the 82,648 men included in the main analyses, 5.9% reported having an FOBT alone, 44.9% reported having a PSA test alone, 18.7% reported having both tests, and 30.6% reported having neither test within the previous 2 years. Of the 96,711 women included in the main analyses, 3.2% reported having an FOBT alone, 56.0% reported having a mammogram alone, 16.2% reported having both tests, and 24.7% reported having neither test within the previous 2 years. The prevalence of test use weighted for age and region of residence among men in our sample (according to the NSW population in 2006 [37]), was 23.1% for FOBT and 62.3% for PSA tests. Among women, the weighted prevalence was 17.7% for FOBT and 68.2% for mammography. Table 1 shows the distribution of each socioeconomic, demographic and health-related characteristic by each test combination (FOBT, PSA test, both tests, or neither test) for men and Table 2 shows the distribution of each socioeconomic, demographic and health-related characteristic by each test combination (FOBT, mammogram both tests, or neither test) for women.

### Multinomial logistic regression

**Figure 1:**
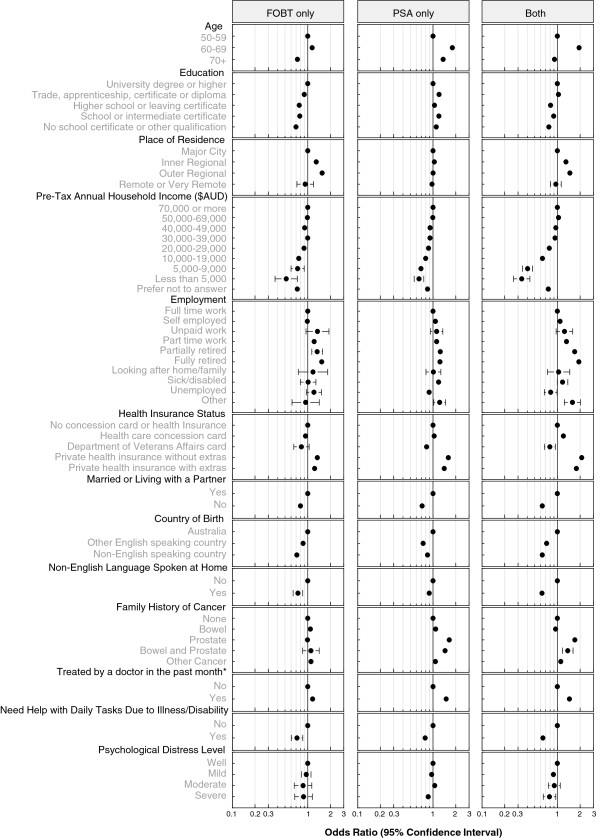
Odds ratios and 95% confidence intervals for receipt of faecal occult blood test (FOBT) alone, prostate specific antigen (PSA) test alone, or both tests for men in the previous 2 years compared to those who reported having neither test in the 45 and Up study.

**Figure 2:**
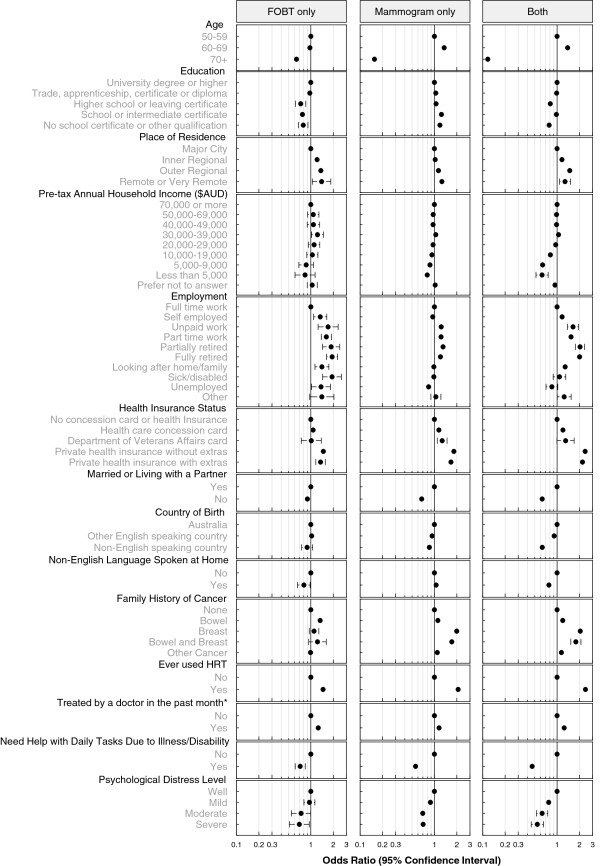
Odds ratios and 95% confidence intervals for receipt of faecal occult blood test (FOBT) alone, mammogram alone, or both tests for women in the previous 2 years compared to those who reported having neither test in the 45 and Up study.

Figure 1 shows the odds ratios for receipt of FOBT alone, PSA test alone, or both tests compared to men who reported having neither test in the previous 2 years. The model included all the factors listed in Table 1 and all factors were significantly associated with screening test use (*p* < .0001). Figure 2 shows the odds ratios for the receipt of FOBT alone, mammography alone, or both tests compared to women who had neither test in the previous 2 years. The model included all the factors listed in Table 2 and all factors were significantly associated with screening test use (*p* < .0001). In order to summarise the results presented in the figures we either highlight the factors with odds ratios (OR) of ~2 or more (or ~0.5 or less) or factors having the largest independent effects.

Overall, the factors with estimated ORs of ~2 or more (or ~0.5 or less) for the receipt of both FOBT and PSA tests among men were age (60–69 vs 50–59 years; OR = 1.94, 95% CI 1.83-2.06), annual household income less than $9,000 (vs. $70,000+; OR = 0.40, 95% CI 0.35-0.47) or $5,000 (OR = 0.34, 95% CI 0.26-0.43), being fully-retired (vs. full-time employment; OR = 1.93, 95% CI 1.79-2.07) and having private health insurance (OR = 2.10, 95% CI 1.96-2.25). For having PSA tests alone, the factors with the largest independent effects were age (60–69 vs 50–59 years; OR = 1.80, 95% CI 1.72-1.89), having a family history of prostate cancer (OR = 1.64, 95% CI 1.54-1.75), having private health insurance (OR = 1.60, 95% CI 1.52-1.68) and being treated by a doctor in the past month (OR = 1.49, 95% CI 1.44-1.55). For use of FOBTs alone among men, the factors associated with the largest independent effects were income less than $5,000 (vs. $70,000+; OR = 0.52, 95% CI 0.37-0.73), living in outer regional areas (vs. major city; OR = 1.55, 95% CI 1.42-1.69), and being fully-retired (vs. full-time employment; OR = 1.53, 95% CI 1.37-1.71.

Among women, the factors with ORs of ~2 or more (or ~0.5 or less) for the receipt of both FOBT and mammograms were age (70+ vs 50–59 years; OR = 0.12, 95% CI 0.11-0.13), use of HRT (OR = 2.41, 95% CI 2.29-2.52), health insurance status: private (OR = 2.38, 95% CI 2.22-2.56) or private plus extras (OR = 2.19, 95% CI 2.02-2.39), family history of breast cancer (OR = 2.05, 95% CI 1.89-2.21), being partially retired (vs. full-time employment; OR = 2.03, 95% CI 1.76-2.35) or fully retired (OR = 2.00, 95% CI 1.84-2.19), and needing help with daily tasks (OR = 0.46, 95% CI 0.41-0.52). For use of mammography alone, the factors with estimated ORs of ~2 or more (or ~0.5 or less) were age (70+ vs 50–59 years; OR = 0.16, 95% CI 0.15-0.17), use of HRT (OR = 2.08, 95% CI 2.01-2.17), and family history of breast cancer (OR = 1.99, 95% CI 1.87-2.12). For use of FOBTs alone among women the factor with the largest independent effect was employment status: fully retired (OR = 1.93, 95% CI 1.63-2.29) or being sick/disabled (OR = 1.93, 95% CI 1.44-2.58) compared to women in full-time employment.

The estimated odds ratios changed less than 10% when we compared the full dataset with a complete case dataset, and also when comparing the full dataset with a sample that excluded men who had ever had an enlarged prostate.

## Discussion

The 45 and Up Study is the largest population-based cohort study in Australia and the results presented here, using uniform outcome measures, suggest that there are different factors related to cancer screening depending on the type of test. Specifically, among women, having mammography alone was associated most strongly with age and well known health-related factors such as HRT use and family history of breast cancer, whereas having a FOBT alone was associated most strongly with being retired or sick/disabled. Among men, having a PSA test alone was associated most strongly with age, family history of prostate cancer, having private health insurance, and being treated by a doctor in the past month, whereas having a FOBT alone was associated most strongly with higher income, living in outer regional areas and being retired. The factors included in our analysis were factors that have been found to be associated with cancer screening in the past [11,14-19], and indeed, all these factors were significantly related to cancer screening in our study. Only one other study has directly compared cancer screening modalities in Australia and that study, restricted to men, also found that PSA testing was more common than bowel screening and that having private health insurance, living with a partner and being white and older were associated with any screening [26].

In Australia, even though national guidelines have advocated bowel cancer screening since 1999 for asymptomatic people from the age of 50 years, a freely available program for bowel cancer screening has only been available to people turning 50 and 55 from 2006 and those turning 50, 55 and 65 from 2008. Prior to 2006, FOBTs were available from 1982 via the Rotary program “Bowelscan” which is run annually throughout most states of Australia at a nominal cost to the individual. In contrast, free population screening for breast cancer has been offered to women aged 50–69 since 1992 and has been widely advocated. PSA testing is not currently recommended as a population-based screening tool for prostate cancer, but is subsidised by the government, has had considerable media attention and is an easy test to administer. Thus, given the circumstances surrounding access to each of these tests in Australia, it is not surprising that FOBT use overall was lower than that for mammography and PSA test use and that having each test is associated with different socioeconomic and demographic factors.

A number of studies have proposed that screening for one cancer type is a potential “teachable moment” for screening for another cancer type, especially given that those having mammograms or PSA tests may be a group that are aware of, and interested in, the benefits of the early detection of cancer [27-29]. The breast cancer screening program in Australia is relatively successful (in terms of uptake and reducing breast cancer mortality [38]), and even though the evidence in favour of PSA testing as a screening tool for prostate cancer remains controversial, many men are being tested regardless. We observed in the 45 and Up Study that 72% of women had a mammogram in the previous 2 years and 63% of men had a PSA test. This suggests that bowel screening rates have the potential to increase markedly from the 22% reported here if promoted through existing health networks. Indeed, in a previous report, we found that 45 and Up Study participants were more than twice as likely to have a FOBT if they had also had a mammogram or a PSA test [30]. Currently, the cancer screening programs in Australia are not integrated. However, decision aids that provide information regarding the harms and benefits of each test side by side may increase the uptake of FOBT – especially for men, since positive messages about screening seem to be deeply ingrained for PSA testing [39].

Socioeconomic factors, especially income, had the strongest effects on being screened for two cancer types. Specifically, those reporting low income and to a lesser degree, education, were less likely to have had two tests. The national monitoring reports of the screening programs, which are based on area-level socioeconomic classification, report lower participation for low socioeconomic sub-groups in the bowel cancer screening program but less so in the breast screening program [9,12,15]. Factors associated with PSA test use have not been widely explored, but one study in Australia found that socioeconomic factors were not strongly associated with PSA test use [18]. We found that low income groups were less likely to have had a FOBT alone, but not a mammogram alone and to a lesser degree, PSA tests alone. Educational status was related to use of FOBT in both men and women, as well as the combined use of FOBT and PSA test/mammography, but was not related to PSA test or mammogram use alone. This finding suggests that low levels of education were specifically related to low levels of bowel cancer screening. Previous studies have demonstrated that social inequality is a factor for the formation of attitudes towards screening [40,41]. For example, one study has shown that people with low socioeconomic status (SES) viewed bowel cancer screening as less beneficial and more frightening than those with higher SES [41]. Thus, when cost and access are not obvious barriers to cancer screening, encouraging people in low socioeconomic groups to participate in the bowel cancer screening program may require targeted promotional campaigns aimed at addressing negative attitudes towards cancer screening.

Interestingly, living in regional and remote areas was not a barrier to cancer screening in our study. Indeed, people in regional and remote areas were *more* likely to be screened for bowel and breast cancer than those living in a major city (there was no variation in the use of PSA tests alone by place of residence). Australia is a vast country and there are health inequalities across geographic locations, especially in areas of geographic isolation, and a number of reports have demonstrated that people in regional areas have poorer survival from cancer than those in major cities [42,43]. However, our study is in concordance with others showing that both mammogram and FOBT uptake via the national programs is higher in rural areas than in the city [12,15,44]. This suggests that geographic differences in cancer survival are possibly due to differences in access to treatments and/or to socioeconomic discrepancies in survival rather than to delayed diagnosis. Higher levels of cancer screening in regional areas could possibly be due to a greater level of community strength and engagement in regional areas, leading to a greater community awareness of cancer screening. For example, the Breast Screening Program in NSW is largely operated out of a mobile van that travels from town to town. This event is likely to be much more salient in a small town with a single central shopping centre than in the urban sprawl of the city, and indeed, in some Australian cities the program is run entirely from clinics. Thus, the presence of the mobile van may facilitate a level of awareness of the program in rural and regional areas that may be lacking in urban areas. Moreover, limited research has shown that community strength is greater in rural Australia than in the cities [45], and higher levels of social integration are positively related to cancer screening uptake [46]. It is also possible that, in the case of bowel screening, people living in major cities are more likely to have a colonoscopy than those in regional areas where accessibility to colonoscopy clinics is more limited (although not explored here).

Those who were retired, in part-time work or in unpaid work were more likely to be screened than those in fulltime work. This result is consistent with studies showing that often people state that lack of time is a reason for not participating in bowel screening [47,48]. That is, those in fulltime work have less free time to be screened. There was very little variation in screening uptake among those who reported their employment status as sick/disabled or unemployed compared to those in fulltime work. However, both men and women who reported needing help with daily tasks because of illness or disability had low levels of screening test use. This may be explained in terms of access issues and the ability to get to a mammography clinic/doctor, or to perform a FOBT at home. Alternatively, it may be the case that these participants have more immediate health problems, possibly a shorter life expectancy, and have therefore made a reasonable decision that the small long-term benefits of screening are not relevant for them. However we also found that those who reported being treated by a doctor for a major illness in the past month were significantly more likely to have been screened than those who had not been treated in the past month. This finding is in line with previous reports showing that routine visits to a doctor are associated with cancer screening [29,40,49-51], and highlights the important role for general practitioners in cancer testing, even though they may not be responsible for administering the tests (i.e. in the case of FOBT and mammography) [48,52-56].

This study had a number of limitations. Firstly, cancer screening history was derived from self-report, however a meta-analysis of validation studies on self-reported cancer screening use in the USA found that self-reported versus documented history of screening had reasonably high sensitivity (0.78 for FOBT, 0.95 for mammography, 0.71 for PSA) and specificity (0.90 for FOBT, 0.61 for mammography, 0.73 for PSA) [57]. Secondly, we were not able to identify participants who had a PSA test for monitoring, rather than screening purposes, however by excluding men who had ever had prostate cancer we were able to eliminate most of these. Additionally, when we excluded men who had ever had an enlarged prostate from the sample, the observed odds ratios changed by less than 10%. Finally, our results may not be representative of the general population because cohort study participants tend to be healthier and more health conscious than non-participants [58] and the participation rate was only 18%. However, like most long-term cohort studies, The 45 and Up Study is designed to provide sufficient heterogeneity for valid comparisons within the cohort, rather than specific estimates of prevalence of exposure in the population [59]. Previous reports have demonstrated that The 45 and Up Study cohort has sufficient spread in the responses to questionnaire items for internal comparisons [31] and that exposure-outcome relationships are very similar to those from a survey of a representative sample from the same population [32]. Potential bias resulting from the “healthy cohort” effect, if it is present, generally leads to more conservative results due to reduced representation of population groups who have more extreme health behaviours, such as those who are mentally or physically ill or marginalised for some other reason. Hence, the associations observed between sociodemographic characteristics and screening may be somewhat closer to the null than might be seen among comparisons of community members who do not tend to participate in studies of this type. Caution must also be exercised when interpreting any negative results.

## Conclusions

Overall, this paper adds to the empirical literature on economic and social discrepancies related to cancer screening uptake. Most importantly, our results highlight the fact that many people are being screened for one cancer type when they could be screened for two. Strategies aimed at using one test as a ‘teachable moment’ for promoting another test may help close the gap in socio-demographic discrepancies in cancer screening to a certain extent.

## Abbreviations

CI: Confidence intervals; FOBT: Faecal occult blood test; HRT: Hormone replacement therapy; NSW: New South Wales; OR: Odds ratio; PSA: Prostate specific antigen; SES: Socioeconomic status.

## Competing interests

The authors have no competing interests to declare.

## Authors’ contributions

MW had overall responsibility for the design of this study, data management, statistical analysis and drafting the paper. MC, DS, and DO’C made substantial contributions to the conception and design of the study, interpretation of the data, and critically revised the manuscript drafts. FS and GS made substantial contributions to the conception of the study and critically revised the manuscript drafts. All authors have given final approval of this version to be published.

## Pre-publication history

The pre-publication history for this paper can be accessed here:

http://www.biomedcentral.com/1471-2458/13/429/prepub
